# Supplementary figures and images for: Root and Shoot Response to Nickel in Hyperaccumulator and Non-Hyperaccumulator Species
Source: Plants (Basel). 2021 Mar 9;10(3):508. doi: 10.3390/plants10030508 (PMC7998499; doi:10.3390/plants10030508)

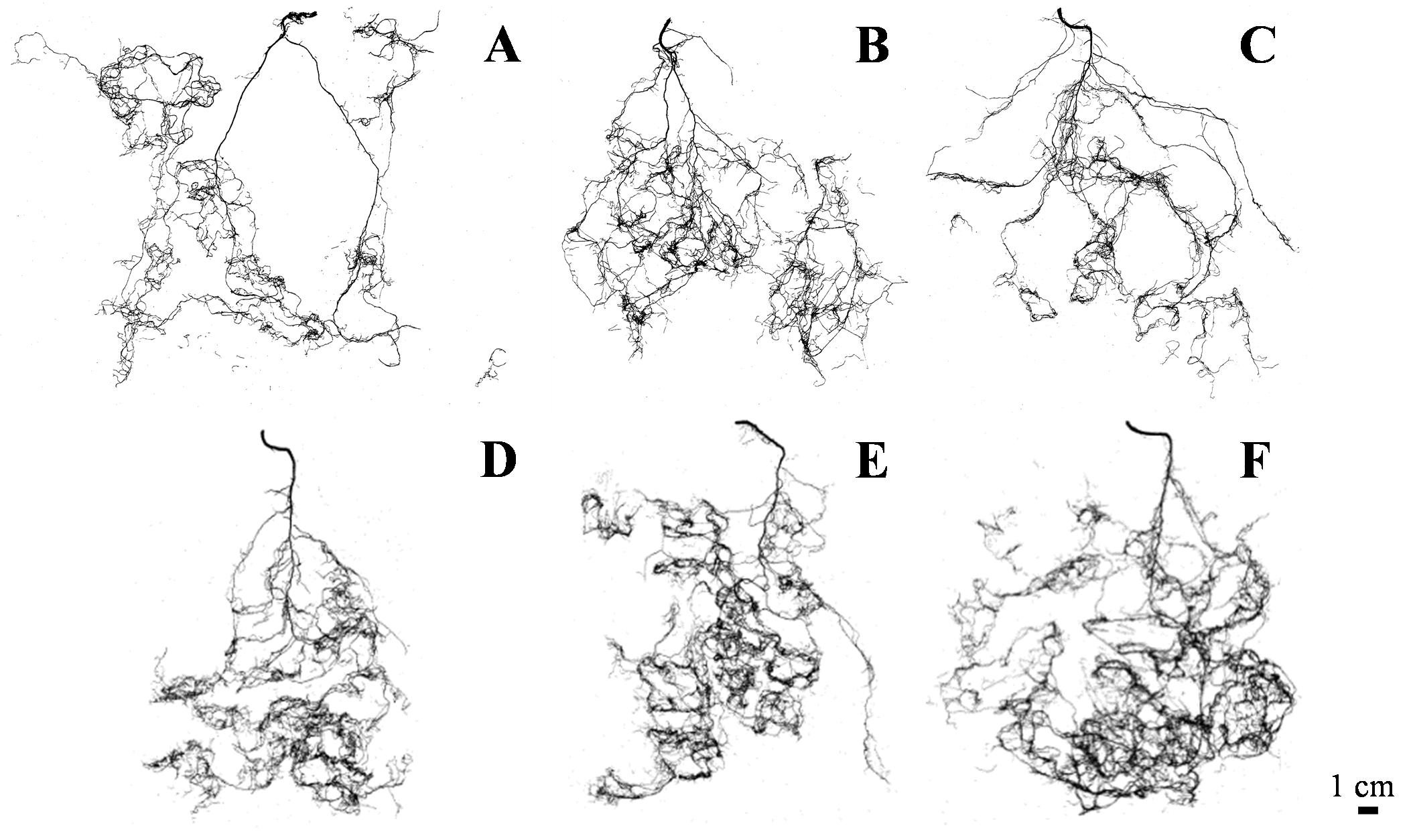

Supplement: Supplementary file 1 [file plants-10-00508-s001.zip › Figure S4.tif]

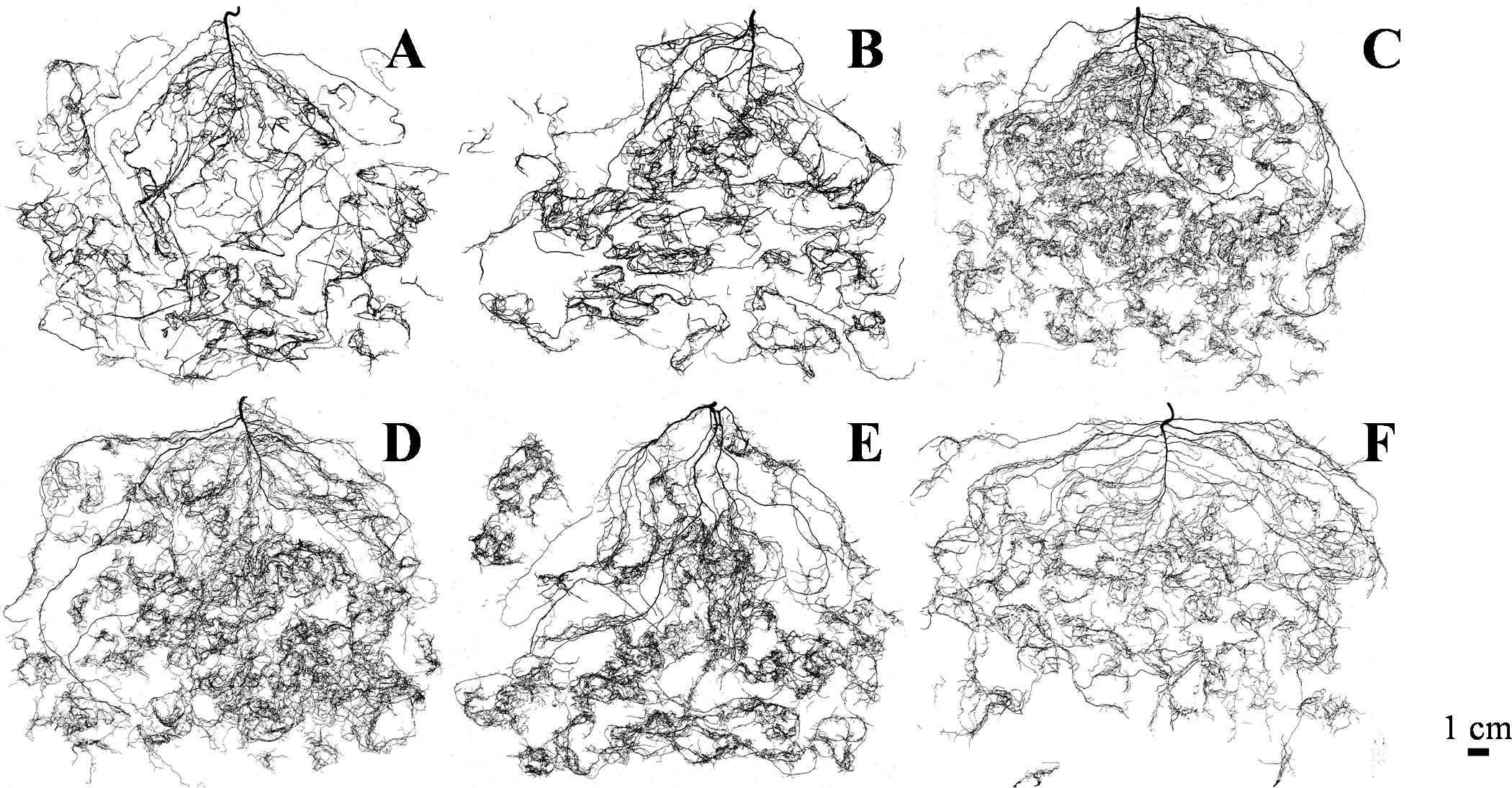

Supplement: Supplementary file 1 [file plants-10-00508-s001.zip › Figure S1.tif]

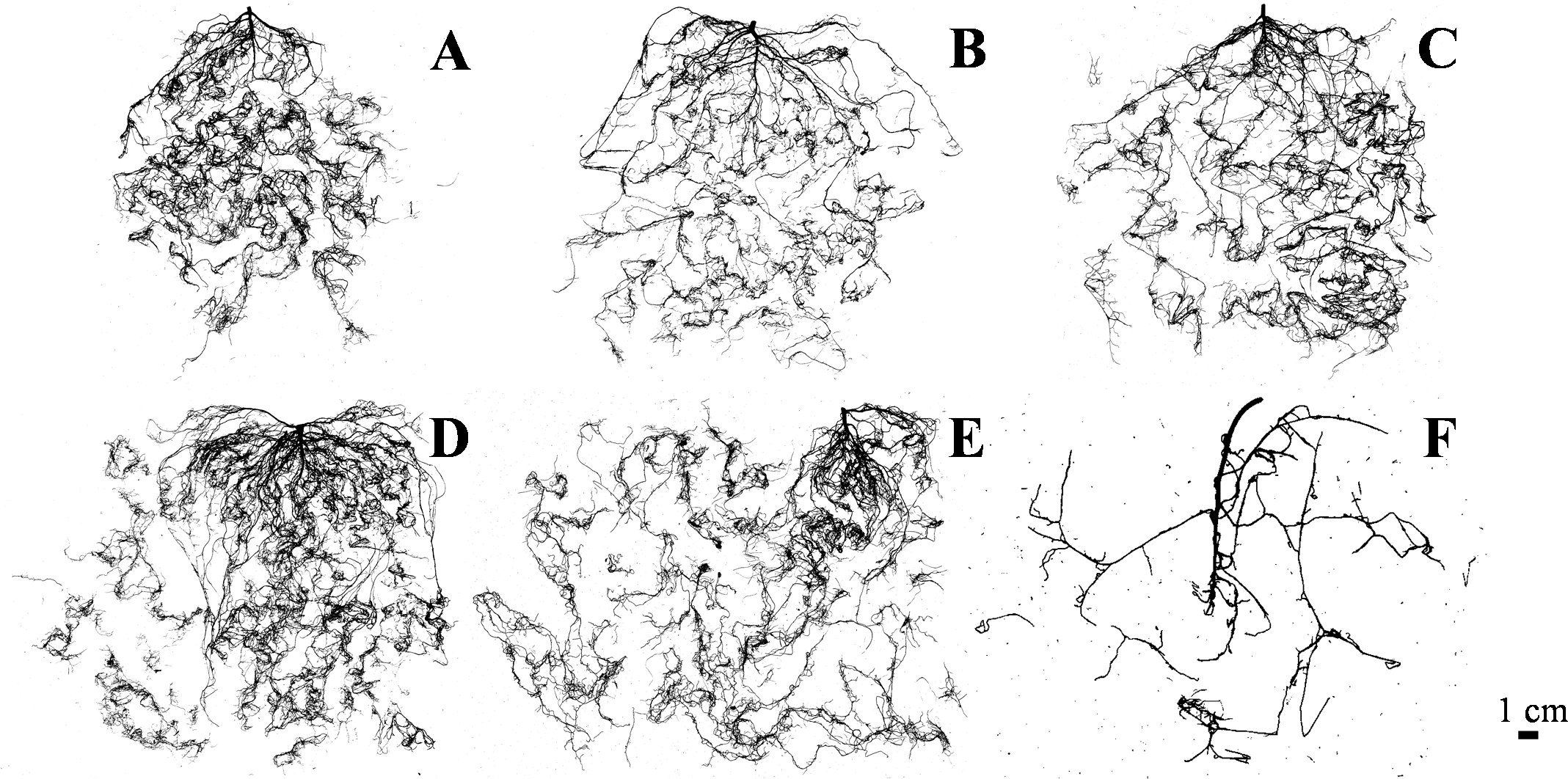

Supplement: Supplementary file 1 [file plants-10-00508-s001.zip › Figure S2.tif]

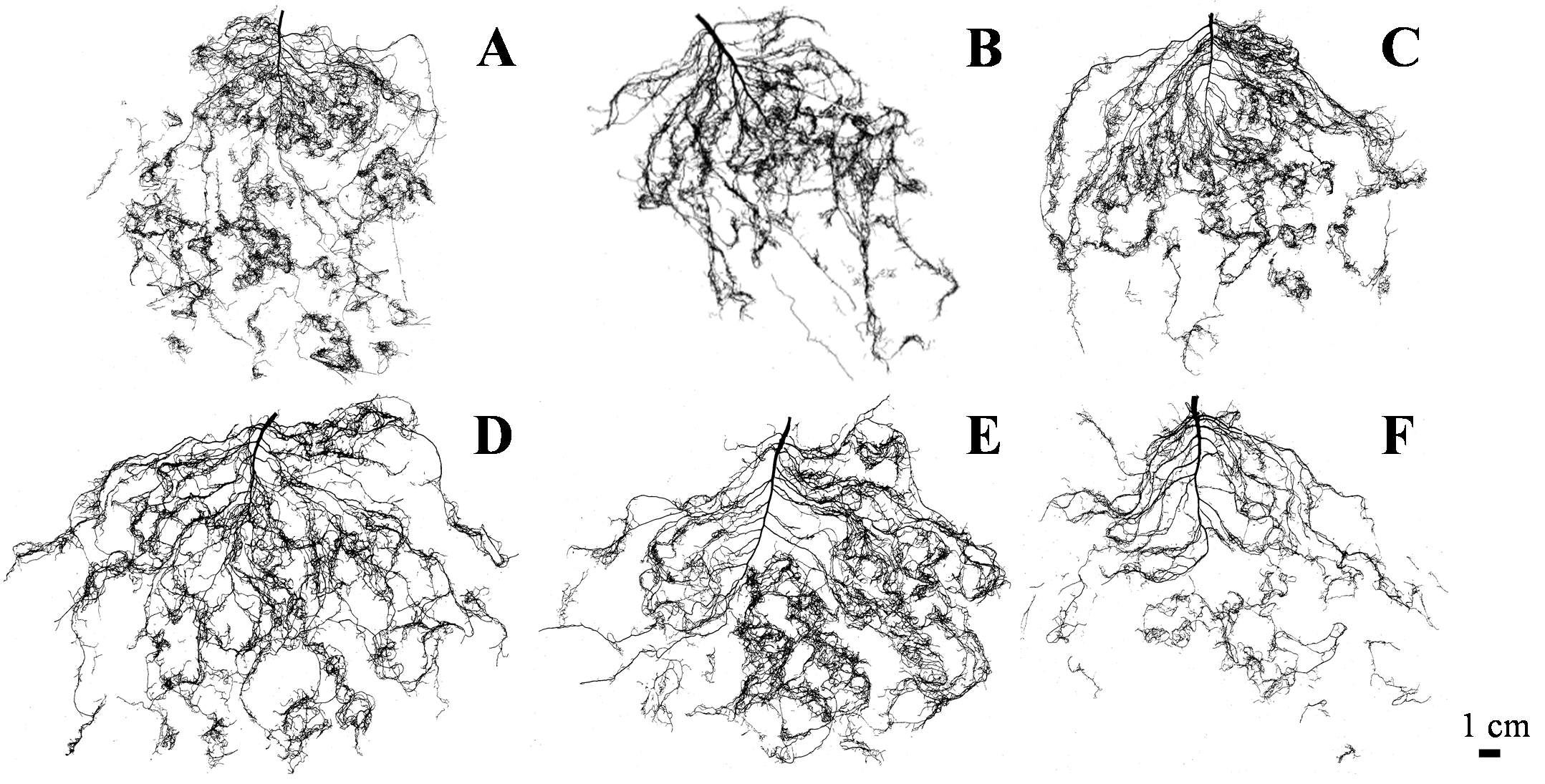

Supplement: Supplementary file 1 [file plants-10-00508-s001.zip › Figure S3.tif]
